# Using an integrated COC index and multilevel measurements to verify the care outcome of patients with multiple chronic conditions

**DOI:** 10.1186/1472-6963-12-405

**Published:** 2012-11-19

**Authors:** Chien-Lung Chan, Huey-Jen You, Hsin-Tsung Huang, Hsien-Wei Ting

**Affiliations:** 1 Department of Information Management, College of Informatics, Yuan Ze University, No. 135 Yuan-Tung Road, Zhongli, Taiwan; 2 Medical Affairs Division, Bureau of National Health Insurance, No.140, Sec. 3, Hsin-Yi Road, Taipei, Taiwan; 3 Department of Health, Taipei Hospital, No. 127, Su-Yuan Rd, Hsin-Chuang District, New Taipei City, Taiwan

## Abstract

**Background:**

The increasing prevalence of multiple chronic conditions has accentuated the importance of coordinating and integrating health care services. Patients with better continuity of care (COC) have a lower utilization rate of emergency department (ED) services, lower hospitalization and better care outcomes. Previous COC studies have focused on the care outcome of patients with a single chronic condition or that of physician-patient relationships; few studies have investigated the care outcome of patients with multiple chronic conditions. Using multi-chronic patients as subjects, this study proposes an integrated continuity of care (ICOC) index to verify the association between COC and care outcomes for two scopes of chronic conditions, at physician and medical facility levels.

**Methods:**

This study used a dataset of 280,840 subjects, obtained from the Longitudinal Health Insurance Database (LHID 2005), compiled by the National Health Research Institutes, of the National Health Insurance Bureau of Taiwan. Principal Component Analysis (PCA) was used to integrate the indices of density, dispersion and sequence into ICOC to measure COC outcomes - the utilization rate of ED services and hospitalization. A Generalized Estimating Equations model was used to verify the care outcomes.

**Results:**

We discovered that the higher the COC at medical facility level, the lower the utilization rate of ED services and hospitalization for patients; by contrast, the higher the COC at physician level, the higher the utilization rate of ED services (odds ratio > 1; Exp(β) = 2.116) and hospitalization (odds ratio > 1; Exp(β) = 1.688). When only those patients with major chronic conditions with the highest number of medical visits were considered, it was found that the higher the COC at both medical facility and physician levels, the lower the utilization rate of ED services and hospitalization.

**Conclusions:**

The study shows that ICOC is more stable than single indices and it can be widely used to measure the care outcomes of different chronic conditions to accumulate empirical evidence. Concentrated care of multi-chronic patients by a single physician often results in unsatisfactory care outcomes. This highlights the need for referral mechanisms and integration of specialties inside or outside medical facilities, in order to optimize patient-centered care.

## Background

A patient is defined as having multiple chronic conditions if he or she has two or more chronic conditions [1], which is a manifestation of multi-comorbidity [2,3]. As aging is accompanied by an increasing prevalence of chronic diseases, the disease pattern of the population in developed and developing countries is shifting from infectious diseases, the highest proportion in the past, to chronic diseases which target an aging population. The increase in the number of elderly people, coupled with the evolution of diseases, has led to a steady increase in the incidence of multiple chronic conditions [4,5]. Chronic patients have, therefore, become the major users of health care systems. In view of this problem, the coordination and integration of care for patients with multiple chronic conditions not only presents a challenge to the health care system [6,7], but has also become an important issue in health policies around the world in the 21st century [8,9]. Compared with patients with a single chronic condition, multi-chronic patients deteriorate faster. They also develop disabilities easily due to medical negligence, such as, drug-drug interaction [1]. Hence, multi-chronic patients require the integration of specialties and continuity of care (COC). This may prevent unnecessary medical services such as repeated medications and examinations, avoid treatment interactions, and obtain a better quality of care [10]. Especially in a modern health care system, which emphasizes the division of professions among specialty carers, the coordination and integration of patient-centered care has become even more important [1-3].

Continuity of care (COC), which has been commonly accepted as a critical factor in enhancing the quality of care for chronic patients, is widely regarded as the basis of primary care and is defined as a form of seamless and connected care provided according to a patient’s needs [11-13]. COC is defined as a health care service that extends over a period of time, during which there is effective and timely exchange of health information between individual medical professionals or within a medical team. COC provides medical facilities with a better understanding of the patient’s medical history. It can strengthen the physician-patient relationship during the course of care and contribute to more effective case management, as well as develop a long-term case monitoring mechanism [13]. Thus, COC is generally regarded to encompass three aspects: (1) Information continuity, i.e. continuity of all previous treatment and care-related medical records; (2) Management continuity, i.e. continuous management of the status of diseases; and (3) Relational continuity, i.e. continuity in the relationship between patients and a single or multi-care provider [14-16]. Previous studies have mainly focused on the relationship between COC and care outcomes, such as the satisfaction of physician-patient relationship, case management and the impact that compliance with doctors’ orders produces on care [17,18].

Some studies have proposed the concept of fixed COC, such as receiving COC from particular physicians [19] or medical facilities [20]. By effectively transmitting medical information, fixed COC can strengthen case management and maintain an effective patient-physician relationship, thereby improving the outcome of medical care [8,20]. Mainous and Gill (1998) discussed the influence of COC on admission rates at different medical facility levels. It was found the physician and medical site (facility) levels produced different results [21]. For multiple chronic conditions, patients need more integrated care from different specialties or a medical team, especially in modern medical systems where there is clear division of specialties. Hence, the study highlights the significance of medical facility levels in COC, which is a particularly important issue when considering the care of multi-chronic patients.

For practical applications, most studies have focused on a single chronic condition and its care outcomes, such as diabetes, asthma and chronic obstructive pulmonary disease [22-26], but few have focused on patients with multiple chronic conditions [1]. However, the status of care for a single chronic condition often leads to other complications (such as diabetes, which often triggers cardiovascular and kidney diseases), so a common medical behavior is to set one chronic condition as the major condition for treatment [1]. Thus, for multi-chronic patients, COC goes beyond the scope of a major chronic condition; it should also consider all chronic conditions (multi-comorbidity) and their care outcomes.

Previous research has demonstrated that a higher COC results in better care outcomes [12,13], such as reducing the frequency of emergency department (ED) visits and hospitalization, while offering better preventative care [18,26,27], better control of chronic diseases [22], and higher patient satisfaction [20,28]. COC emphasizes the process of care, but data collection is difficult [24,29]. While some qualitative studies used a theoretical model, they still lacked empirical evidence [29]. Different indices have been constructed for COC: some emphasize the duration of the patient-physician relationship; some emphasize the frequencies or sequences of physician care, and some focus on physician numbers. Jee and Cabana (2006) conducted a systematic review on COC indices and presented their advantages and disadvantages, but the study does not point out which index is superior [30]. For example, the algorithm for Usual Provider Care (UPC) focuses on the number of physicians visited or the visit ratio of the most frequently visited physicians. But UPC cannot detect whether patients reduce their visits or change physicians frequently [31]. The Continuity of Care Index (COCI) calculates both the total visiting numbers and the number of caregivers, but the calculation is complex [22]. Sequential Continuity of Care (SECOC) can calculate the sequences of change in medical care, but it may not be suitable for non-sequential issues. Previous research has emphasized that there is no consistent and standardized integrated index for COC [29,30], and an individual index cannot present every aspect of COC assessments [24,32-34]. Furthermore, few indices can explain the relationship between the caregivers and care types. Jee and Cabana (2006) have suggested integrating different types of indices into an integrated index or constructing an index with indices of different weights, in order to assess the COC related to different types of medical care [30].

In 1995, Taiwan implemented the National Health Insurance (NHI) system, which provides near-universal coverage, with more than 92% of medical care institutions in Taiwan providing NHI medical services [35,36]. Like many Asian and European countries, the health care system in Taiwan lacks family physicians and an effective referral mechanism. The general public can freely select a medical site (e.g., clinic or hospital), regardless of the severity of the disease [17,26]. The different types of medical services affect the way the public seek medical treatment, which is considerably different from the situation in Europe and America. Western countries that promote integrated care mainly operate under a health care system with family physicians and referral mechanisms. The family physician is the first stop for the public. The physician diagnoses the patient and provides suitable treatment; and if necessary, the patient is referred to a specialist hospital or a large hospital [37]. By contrast, all hospitals in Taiwan, whatever the size and scale, have a comprehensive care service with different specialist physicians and provide primary care services, as well as outpatient and inpatient services for emergency and critical care [27]. Due to the accessibility to medical care in Taiwan, the National Health Research Institutes (NHIR) can easily collect longitudinal data of care outcomes and case management of chronic patients at both medical facility and physician levels.

Because Taiwan’s health insurance system has a high coverage and there is ease of access to medical care, Taiwan provides an ideal environment for empirical study on COC. This study aims at constructing an integrated COC (ICOC) index, and verifying the association between ICOC and care outcomes for different scopes of chronic conditions (all chronic conditions and major chronic condition) at physician level and medical facility level.

## Methods

### Data source

Taiwan launched a mandatory, single-payer National Health Insurance (NHI) program in 1995, which offers a comprehensive and uniform benefits package to provide universal coverage and convenient access to health care services. About 23 million people were enrolled in the NHI program in the years 2005–2009 (covering more than 99% of Taiwan’s citizens), and most hospitals and health care providers are contracted by the NHI system (more than 92% of all medical facilities). The contract western medical institutions are 4.8 per 10,000 beneficiaries (the number of western medical institutions in 2010 was 11,107), and contract western medical doctors are 16.8 per 10,000 beneficiaries (the number of western medical doctors in 2010 was 38,908) [35]. For the purpose of research and policy assessment, the National Health Insurance Bureau (NHIB) collaborated with the National Health Research Institutes (NHRI) to establish a nationwide research database, and routinely transfers relevant NHI administrative data into research databases [35]. The study is a retrospective cohort study. The source of the data was the Longitudinal Health Insurance Database (LHID 2005), a subset of the National Health Insurance Research Database (NHIRD) compiled by the National Health Research Institutes (NHRI) under the National Health Insurance Bureau (NHIB) in Taiwan. The 2005 Longitudinal Health Insurance Database consists of 1 million subjects in 25 subsets of randomly selected samples from the entire NHI enrollee profiles. Random sampling was conducted for every sub-set of 40,000 subjects enrolled in the NHI at the end of 2005. The LHID 2005 database was thus considered to have representative power of the national population [38]. The dataset used contains outpatient and inpatient claims data from 2004 to 2009, with the details of each visit recorded, while the registry file of beneficiaries was processed in order to access the demographic data. Permission to use the research data in this study has been approved by an administrative process in NHIB, and the research data has undergone data encryption and privacy protection by IT department.

**Figure 1:**
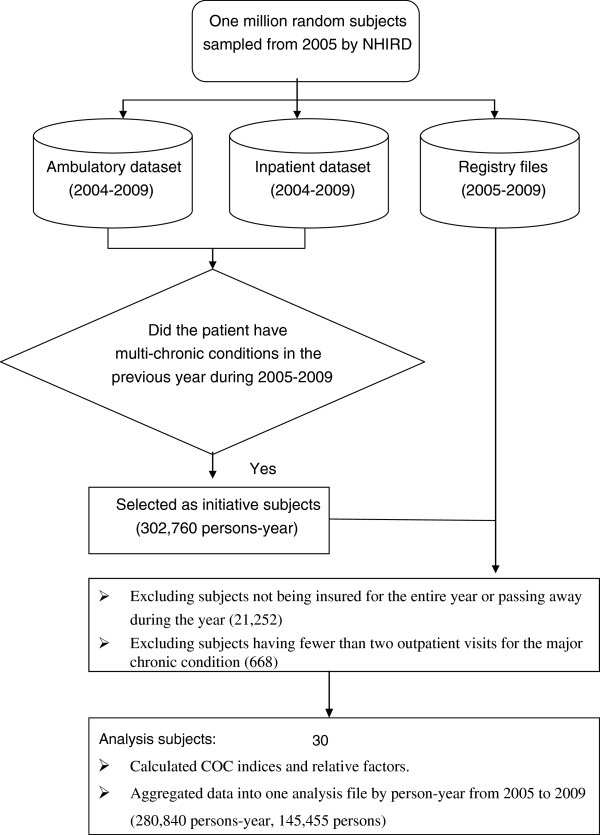
**Flowchart of subject selection and data extraction** .

Many studies define chronic conditions using major diagnostic categories (MDCs) [1,34]. In this study, we used the main diagnoses of five MDC codes from the medical records. The chronic conditions were defined by MDCs based on NHI specified chronic disease ICD-9-CM codes, including MDCs 1–13, 15–21 and 24 (MDC 21 only includes the oil of PCBs (polychlorinated biphenyls) poisoning (see Additional file 1) [39]. The criteria for a chronic condition in the respective MDCs were defined as patients who had one or more hospitalization events, or two or more outpatient visits for the same MDC, and whose period of outpatient visits and medication was spread over 180 days or more per observed year. The diagnosis of two or more chronic conditions in the previous year was taken as the definition for multiple chronic conditions and such patients were selected as the initiative analysis subjects. Non-Taiwanese enrollees were excluded from the study. In total, 302,760 subjects (persons-year) were included in the first phase from 2005 to 2009. A total of 21,252 subjects were excluded as they had not been insured for the entire observed year or had passed away during the observed year, and a total of 668 subjects were excluded because they had less than two outpatient visits for the same major chronic condition. As a result, 280,840 subjects (persons-year) were analyzed in this study. Figure 1 shows the flowchart of subject selection and data extraction. The data were compiled into annual personal records from 2005 to 2009, which included demographic characteristics and health status, as well as any level of medical care and COC measurements of chronic conditions that differed from the previous year.

### Outcome variables

A review of relevant literature on the association between COC indices and care outcomes showed that most studies used the utilization of ED services, hospitalization, or both to measure care outcomes [12,17,18,26,27]; other variables for care outcome included satisfaction, days of hospitalization, mortality and medical cost [1,3,12]. This study used the most popular indices to measure care outcome. The utilization of ED services/hospitalization was set as ‘1’ when a patient used ED services/hospitalization in the observed year, while non-users of ED services/hospitalization were set as ‘0’ for outcome variables.

### Measurement of COC indices

Jee and Cabana (2006) conducted a systematic literature review on COC measurements and summarized the popular COC types and indices. Based on their findings, we selected three types of COC indices corresponding to the most commonly-used information in the claims data: density, dispersion and sequence [16,17,27,31]. The respective indices are the Usual Provider of Care (UPC), Continuity of Care Index (COCI) and Sequential Continuity of Care (SECOC). The values ranged between 0 and 1, with higher values indicating a higher COC.

The general formulas are as follows:

1. Density: Usual Provider Care (UPC) – the index can be used to identify the most frequently visited physician. UPC measures the highest density (frequency) of a patient’s visits to a physician, with visits to the same physician being quantified as the number or percentage of visits over a defined time period.

(1) $$UPC=\frac{\begin{array}{c} \max\\ 1\;\leq \;j\;\leq \;M \end{array}\left(nj\right)}{N}$$

2. Dispersion: Continuity of Care Index (COCI) – the index measures the dispersion of visits, which quantifies the number or percentage of visits to distinct providers.

(2) $$COCI=\frac{\sum_{j=1}^{M}n_{j}^{2}-N}{N\left(N-1\right)}$$

3. Sequence: Sequential Continuity of Care (SECOC) – the index sequentially measures the various physicians visited in the order in which different providers are seen.

(3) $$SECOC=\frac{\sum_{i=1}^{N-1}Si}{N-1}$$

Where N = total number of visits (excluding ED visits)

n_j_: number of visits to the *j*th different provider, j = 1, 2… M

M = number of potentially available providers

s_i_: if the *i*th visit and the subsequent *(i + 1)*th visit are to the same provider then s_i_ =1, and s_i_ = 0 otherwise.

As different types of COC indices differ in the nature of measurement, they may produce different results when used to measure the COC of varying levels of medical care [17,30]. In light of this, we attempted to construct a care outcome association model using Principal Component Analysis (PCA) as the basis for integrating the COC indices. We identified three commonly-used COC indices (UPC, COCI and SECOC) [17,31], determined their weights and calculated the weighted means of the variables for each type of index, and produced the integrated COC index (ICOC). The general formula is as follows:

(4) $$ICOC=\left(\beta_{1}UPC\;+\;\beta_{2}COCI+\beta_{3}SECOC\right)/\left(\beta_{1}+\beta_{2}+\beta_{3}\right)$$

Where (β_1_,β_2_, β_3_) is the first principal component Eigenvector of PCA result.

The ICOC was used to measure two different chronic condition scopes at physician and medical facility levels. The two chronic condition scopes are: all chronic conditions (all the chronic conditions that the patient was found to have during the period of observation), and the major chronic condition (the chronic condition resulting in the highest number of outpatient visits during the observed period). The two scopes were each measured for their COC outcome at physician level and medical facility level. Physician level refers to individual providers of medical care, while medical facility level refers to the medical care institution.

### Other relevant factors

As demographic characteristics and health status are important factors that affect care outcome, they have often been used by researchers of COC as control variables to observe the impact of COC. We used age, sex and income status [27,40] as important demographic factors to analyze and extract data from the registry files. Low-income status is defined as patients whose family income is less than the minimum insured amount of NT$ 18,200 (US$ 1 = NT$ 30) [36].With regard to the health status factors, many researchers have incorporated the Charlson Comorbidity Index (CCI) [34,40,41] (containing 17 categories of comorbid conditions defined by the ICD-9-CM codes) and the number of chronic conditions (NCC) [22,27] in their study. Both CCI and NCC were included in this study. This study also considered some well-known variables which are associated with health status or need. We also considered whether patients had disabilities or catastrophic illnesses. According to the Ministry of Internal Affairs of Taiwan, 11 kinds of disability (including visual and hearing impairments) are listed in the NHI (see Additional file 2) [42]. Catastrophic illnesses were defined by the NHI based on the ICD-9-CM codes and include 31 items of major injuries and diseases (such as cancer, rare diseases and end-stage renal diseases) [43]. To understand the actual impact of care outcomes, we processed the demographic characteristics and health status factors by year. Then the factors were extracted and inserted into eligible subjects by persons-year to be used as control variables.

### Statistical methods

We used descriptive analysis to examine the baseline differences in the person-year utilization rate of ED services/hospitalization, and Chi-square tests to determine the differences of distribution between the groups of user and non-user for each relevant factor. To understand the explanatory ability of the ICOC index and factor correlations (factor loading) of the PCA results, we presented the variable explanatory of the first principal component of PCA, and used Correlation Coefficients (CC) to assess the factors correlation between ICOC and others COC indices.

To establish the relationship between the different ICOC and outcomes and eliminate any bias arising from the differences in the demographic characteristics and the subjects’ health conditions, this study regarded the subjects using ED services/hospitalization as the target/experiment group. We used Propensity Score Matching (PSM) to select matching subjects among other eligible subjects. PSM has been commonly used in the field of healthcare [44-48]. This study constructed PSM using stepwise logistic regression with factors of demographic characteristics and health status. The 5 to 1 digit matching method was then used to match patients who had used ED services or hospitalization to not-users. Moreover, with the other relevant factors under control, this study used T-tests to evaluate the differences of ICOC between the groups.

A linear model to compare ICOC with the three popular individual indices was then constructed. The model also evaluated the association between the measurements of ICOC and other COC indices for different aspects of care. Since the outcome variables are binary data, and the data of some subjects might have been repeatedly calculated during the observed period, we applied a generalized linear model with a logit link function and binomial distribution to conduct the analysis. In addition, we adopted the Generalized Estimating Equations (GEEs) method to fit the model for data analysis, as GEE is a practical method with reasonable statistical efficiency. To control the impact of the demographic characteristics and health status on the utilization of ED services/hospitalization, and to avoid model fitting un-convergence caused by using so many factors in the model construction, we used the demographic propensity need (DPN) score calculated by PSM. The DPN score represents the utilization risk of ED services/hospitalization for different demographic characteristics and health status [46,49]. Thus, this study regarded the demographic characteristics and health needs as a synthesized proxy variable. We simplified the model and its fitness is improved as compared with traditional models, which do not have model fitting un-convergence. Finally, we constructed a model of ICOC and others COC indices. By adjusting and controlling other variables, the odds ratio and p value of statistical difference showed the goodness of the model fitting. Our analysis was performed using statistical analysis software: SAS version 9.2 (SAS Institute Inc., Cary, NC).

We used a vast amount of data from national representative samples in the analysis. As we solely used statistical significance testing to compare the differences, our results were influenced by the large sample size, and were thus always statistically significant [50]. Therefore, our analysis accepted a significance level of 0.001 in the interpretation of the odds ratio.

## Results

### Demographic statistics

The subject characteristics and the distribution of ED services and hospitalization utilization rates are listed in Table 1. Overall the utilization rate of ED services and hospitalization were 4.99% and 21.36%. The differences in the utilization rate of ED services and hospitalization between the groups for each relevant factor are statistically significant, as they were under the 0.001 significance level. The demographic characteristics showed that, compared to non-users, users were more likely to be in the ≥ 65 years age group (ED: 59.35% vs. 38.57%, HP: 54.20% vs. 35.64%), predominantly male (ED: 51.44% vs. 45.04%, HP: 50.21% vs. 44.04%) and of low income (ED: 3.54% vs. 1.62%, HP: 2.86% vs. 1.41%). In terms of health status, users were more likely to be disabled (ED: 35.71% vs. 15.95%, HP: 30.38% vs. 13.28%), with more catastrophic illnesses (ED: 31.11% vs. 15.39%, HP: 31.40% vs. 12.04%), with a Charlson Comorbidiy Index score greater than 3 (ED: 58.73% vs. 23.43%, HP: 52.11% vs. 17.89%), and have more than three chronic conditions (ED: 37.42% vs. 25.09%, HP: 35.28% vs. 23.10%). In summary, the health status of the users was significantly poorer than that of the non-users.

**Table 1:** Subject characteristics and distribution of ED services and hospitalization utilization rates

**Characteristics of study sample**			**Utilization rate of ED services**						**Hospitalization**					
	**Total**		**Non-use**		**Use**		**Testing the consistency of distribution of subjects**		**Non-use**		**Use**		**Testing the consistency of distribution of subjects**	
	**Subjects**	**Rate (%)**	**Subjects**	**Rate (%)**	**Subjects**	**Rate (%)**	***X***^**2**^**p value (Sig. level)**		**Subjects**	**Rate (%)**	**Subjects**	**Rate (%)**	***X***^**2**^**p value (Sig. level)**	
**Utilization rate of ED services**									**Hospitalization**					
Total	280,840	100	266,817	100	14,023	100			220,856	100	59,984	100		
Age														
≤ 18	21,591	7.69	21,168	7.93	423	3.02	0.0000 *		19,611	8.88	1,980	3.3	0.00000 *	
18 ~ 64	148,015	52.7	142,738	53.5	5,277	37.63			122,522	55.48	25,493	42.5		
≥ 65	111,234	39.61	102,911	38.57	8,323	59.35			78,723	35.64	32,511	54.2		
Sex														
Male	127,387	45.36	120,173	45.04	7,214	51.44	0.0000 *		97,268	44.04	30,119	50.21	0.00000 *	
Female	153,453	54.64	146,644	54.96	6,809	48.56			123,588	55.96	29,865	49.79		
Income status														
Non low-income	276,017	98.28	262,491	98.38	13,526	96.46	0.0000 *		217,750	98.59	58,267	97.14	0.00000 *	
Low-income	4,823	1.72	4,326	1.62	497	3.54			3,106	1.41	1,717	2.86		
Disability status														
No	233,285	83.07	224,270	84.05	9,015	64.29	0.0000 *		191,523	86.72	41,762	69.62	0.00000 *	
Yes	47,555	16.93	42,547	15.95	5,008	35.71			29,333	13.28	18,222	30.38		
Catastrophic illnesses														
No	235,420	83.83	225,759	84.61	9,661	68.89	0.0000 *		194,269	87.96	41,151	68.6	0.00000 *	
Yes	45,420	16.17	41,058	15.39	4,362	31.11			26,587	12.04	18,833	31.4		
Charlson Comorbidity Index score														
0	90,919	32.37	89,451	33.53	1,468	10.47	0.0000 *		82,918	37.54	8,001	13.34	0.00000 *	
1 ~ 2	119,165	42.43	114,846	43.04	4,319	30.8			98,437	44.57	20,728	34.56		
≥ 3	70,756	25.19	62,520	23.43	8,236	58.73			39,501	17.89	31,255	52.11		
														
2	208,663	74.3	199,887	74.92	8,776	62.58	0.0000 *		169,842	76.9	38,821	64.72	0.00000 *	
≥ 3	72,177	25.7	66,930	25.09	5,247	37.42			51,014	23.1	21,163	35.28		

Note: ‘*’ indicates statistical significance under P ≤ 0.001. ED: Emergency Department.

### Integration of COC indices

In this study three commonly-used COC indices, UPC, COCI and SECOC, were integrated into the ICOC using PCA. The explanatory variable of the first principal component of PCA for the measurements of the major chronic condition at both physician and medical facility levels and that of the all chronic conditions at medical facility level were all greater than 90% (94.37%, 94.23% and 92.20%, respectively), while that of all chronic conditions at physician level was 88.70% (results not shown). The descriptive statistics and factors correlation between ICOC and each individual index are shown in Table 2. Different aspects of the ICOC index are highly and positively correlated to the individual COC index with PCC > 0.9, which demonstrates the reliability of the ICOC index in representing the single COC indices. It also maintains the original meaning of the COC index: the higher the index, the higher the continuity. Whether at medical facility or physician level, the measurements of the COC indices for all chronic conditions were lower than those for the major chronic condition, especially at physician level. This demonstrates the need for multi-specialty care among multi-chronic patients, as well as the necessity for COC measurement in the COC of multi-chronic patients at both medical facility and physician levels.

**Table 2:** Descriptive statistics and factors correlation between ICOC and each individual index

			**Mean (Std Dev)**	**Min**	**Max**	**Correlation with ICOC**	
COC index of medical facility level							
	All chronic conditions						
		Density-UPC	0.6809 (0.2088)	0.0903	1.0000	0.9621	
		Dispersion-COCI	0.5561 (0.2577)	0.0000	1.0000	0.9780	
		Sequence-SECOC	0.6071 (0.2623)	0.0000	1.0000	0.9415	
		Integrated Index-ICOC	0.6099 (0.2356)	0.0359	1.0000	1.0000	
	Major chronic condition						
		Density-UPC	0.9037 (0.1609)	0.1032	1.0000	0.9724	
		Dispersion-COCI	0.8495 (0.2352)	0.0000	1.0000	0.9900	
		Sequence-SECOC	0.8897 (0.1933)	0.0000	1.0000	0.9426	
		Integrated Index-ICOC	0.8772 (0.1958)	0.0391	1.0000	1.0000	
COC index of physician level							
	All chronic conditions						
		Density-UPC	0.5312 (0.1966)	0.0417	1.0000	0.9457	
		Dispersion-COCI	0.3713 (0.2169)	0.0000	1.0000	0.9665	
		Sequence-SECOC	0.4375 (0.2367)	0.0000	1.0000	0.9177	
		Integrated Index-ICOC	0.4433 (0.2051)	0.0126	1.0000	1.0000	
	Major chronic condition						
		Density-UPC	0.7783 (0.2266)	0.0417	1.0000	0.9693	
		Dispersion-COCI	0.6689 (0.3094)	0.0000	1.0000	0.9877	
		Sequence-SECOC	0.7380 (0.2797)	0.0000	1.0000	0.9506	
		Integrated Index-ICOC	0.7225 (0.2684)	0.0156	1.0000	1.0000	

Note: PCC, Pearson Correlation Coefficients.

### Difference of ICOC in utilization status

Table 3 shows the ICOC differences between the user and non-user groups in terms of utilization rates of ED services and hospitalization. At both levels the average ICOC value of the user group for major chronic conditions, for either the utilization rate of ED services or hospitalization, was significantly lower than that of the non-user group (*p* ≤ 0.001). For all chronic conditions, the mean ICOC value showed no significant difference for the user group at medical facility for the utilization rate of ED services or hospitalization. Moreover, there were significant differences in the ICOC of all chronic conditions for hospitalization at physician level, while no significant difference was found for the utilization of ED services. Overall, a positive association was found between ICOC and care outcomes, as a comparison of the results showed a significant difference between the user and non-user groups.

**Table 3:** Comparison of the differences of ICOC between group utilization status after PSM

				**Utilization rate of ED service**				**Hospitalization**			
				**Mean (StdDev)**		**T-Test H**_**0**_**:μ**_**No**_ **= μ**_**Yes**_		**Mean (StdDev)**		**T-Test H**_**0**_**:μ**_**No**_ **= μ**_**Yes**_	
						**P-Value (sig. level)**				**P-Value (sig. level)**	
COC index of medical facility level											
	All chronic conditions										
		Total		0.6253 (0.2387)				0.6265 (0.2371)			
		Utilization status									
			Non-use	0.6297 (0.2386)		0.0021		0.6245 (0.2361)		0.0058	
			Use	0.6210 (0.2387)				0.6283 (0.2380)			
	Major chronic condition										
		Total		0.8674 (0.1983)				0.8749 (0.1933)			
		Utilization status									
			Non-use	0.8816 (0.1890)		0.0000 *		0.8869 (0.1850)		0.0000 *	
			Use	0.8531 (0.2062)				0.8636 (0.2000)			
COC index of physician level											
	All chronic conditions										
		Total		0.4237 (0.2003)				0.4288 (0.1985)			
		Utilization status									
			Non-use	0.4229 (0.1945)		0.5350		0.4324 (0.1959)		0.0000 *	
			Use	0.4244 (0.2059)				0.4255 (0.2008)			
	Major chronic condition										
		Total		0.6931 (0.2683)				0.7080 (0.2656)			
		Utilization status									
			Non-use	0.7080 (0.2643)		0.0000 *		0.7274 (0.2613)		0.0000 *	
			Use	0.6782 (0.2715)				0.6896 (0.2683)			

Note: ‘*’ indicates statistical significance under P ≤ 0.001; ED: Emergency Department.

### Association between ICOC and respective outcomes

Based on the results of the GEE modeling, demographic characteristics, health status and ownership of medical site were set as control variables. Table 4 shows the adjusted association between COC and care outcomes among COC indices. At the same time, the model fittings of ICOC and the three original COC indices (UPC, COCI, and SECOC) were compared. The results showed that for the utilization rate of both ED services and hospitalization, ICOC had a lower quasi-likelihood information criterion (QIC), indicating that it has a better model fitting. The modeling also showed that the effects of COC on the utilization rate of ED services are of consistent statistical significance for different scopes of chronic conditions at both levels. By contrast, different COC indices showed varying results in terms of hospitalization. SECOC at medical facility level for all chronic conditions had no significant effect on hospitalization, while UPC and COCI showed a high correlation.

**Table 4:** The adjusted association between COC and care outcomes among COC indices – results of the GEE model

	** Integrated index (ICOC)**		** Density (UPC)**		** Dispersion (COCI)**		** Sequence (SECOC)**	
	**Exp(β)**	**Pr > ChiSq**	**Exp(β)**	**Pr > ChiSq**	**Exp(β)**	**Pr > ChiSq**	**Exp(β)**	**Pr > ChiSq**
Model of Utilization rate of ED service								
Parameter estimate (β)								
COC index of medical facility level								
All chronic conditions	0.800	0.0002	0.779	0.0002	0.804	0.0001	0.856	0.0041
Major chronic condition	0.681	<.0001	0.642	<.0001	0.724	<.0001	0.696	<.0001
COC index of physician level								
All chronic conditions	2.116	<.0001	2.216	<.0001	2.014	<.0001	1.727	<.0001
Major chronic condition	0.591	<.0001	0.544	<.0001	0.636	<.0001	0.646	<.0001
GEE fit criterion								
QIC	101,066		101,090		101,086		101,133	
QICu	101,063		101,087		101,083		101,130	
Model of Hospitalization								
Parameter estimate (β)								
COC index of medical facility level								
All chronic conditions	0.925	0.0212	0.870	0.0003	0.899	0.0008	1.011	0.7063
Major chronic condition	0.630	<.0001	0.601	<.0001	0.683	<.0001	0.641	<.0001
COC index of physician level								
All chronic conditions	1.688	<.0001	1.842	<.0001	1.596	<.0001	1.423	<.0001
Major chronic condition	0.634	<.0001	0.577	<.0001	0.686	<.0001	0.679	<.0001
GEE fit criterion								
QIC	250,260		250,344		250,334		250,390	
QICu	250,257		250,340		250,331		250,387	

Note: QIC: quasi-likelihood information criterion for alternating logistic regressions; QICu: QIC for the unstructured working correlation model.

Different aspects of ICOC had significant impact on the utilization rate of ED services (*p* ≤ 0.001). Notably, when the relevant variables were controlled, an increase in the ICOC at physician level for all chronic conditions demonstrated a high utilization risk of ED services (odds ratio > 1; exp(β) = 2.116), while other ICOC aspects showed the utilization risk of ED services was relatively low (odds ratio < 1). In other words, the higher the COC at physician level for all chronic conditions, the higher the possible utilization risk of ED services. Similar results were found for hospitalization, with a significant effect on all four aspects of ICOC (*p* ≤ 0.001). All measurements at physician level for all chronic conditions also showed that when the unit ICOC increases, the risk of hospitalization is relatively higher (odds ratio > 1; exp (β) = 1.688). In summary, when the degree of ICOC of all other aspects remain the same, the higher the COC at physician level for all chronic conditions, the poorer the outcome. On the hand, the higher the ICOC measurements of the other aspects, the better the result.

## Discussion

Continuity of care is widely believed to be essential for high-quality patient care. Most COC measurements have focused on a single chronic condition [3,11,12,22,51]. In a study of care at different medical sites, Mainous and Gill (1998) found that a high physician continuity of care is better than high facility / low physician or low facility / low physician [21]. In this study we investigated different scopes of chronic diseases, including all chronic conditions and major chronic conditions, and found different care outcomes at physician and medical facility levels. Patients with a major chronic condition received treatment that resulted in good care outcomes at both physician and medical facility levels. For all chronic conditions the care received at medical facility level resulted in good outcomes, but not so at physician level, where outcomes were poor. In other words, for major chronic conditions with the highest numbers of outpatient visits, the higher the continuity of care, the lower utilization rate of ED services and hospitalization, and thus medical resource utilization will be much lower. Medical service providers should establish disease tracking management plans and provide COC services such as patient-centered long-term case management and maintenance of patient-care provider relationship for loyal chronic patients.

This study differs from previous studies in that it used an empirical approach in the study of multi-chronic patients. In doing so we discovered that for multi-chronic patients, all chronic conditions concentrated at medical facility to received care services will lead to a good outcome. By contrast, all chronic conditions concentrated at physician level to received care services may lead to poor outcomes. As chronic diseases are often accompanied by multi-comorbidity, the need for medical care cannot be entirely met only at physician level. This confirmed our view that because multi-chronic conditions require the care of multiple specialties, the needs of multi-chronic patients are difficult to meet at the individual physician level [1,3]. Thus, multi-chronic patients may benefit from a better care outcome if the COC of chronic conditions can take place at integrated facilities or specialties where there is integration of resources and multi-specialities. Because of the increasing need for coordinated care for such patients, different specialists and medical facilities should become integrated in order to enhance the efficiency and efficacy of care, as evidence has shown this to be of benefit [15,24]. Medical service providers need to adjust treatment models and intra or inter-organizational integration or establish a referral mechanism for the collaboration of multiple specialties to enhance the accessibility to multi-specialty services. Several studies have suggested that by doing so, health care outcome can be improved [8,37]. Instead of single-physician care, a patient-centred, multi-specialty-oriented health care structure for multi-chronic patient is very much needed.

Several studies on COC indices have focused on a single index measurement, which can not be applied to all types of medical care [3,30,33]. This study validated the conclusion of previous studies that different patterns of COC may lead to different results, hence the need for the integration of COC indices [27,30]. As ICOC was found to have a better model fitting, we used the respective findings as the basis to put forth our views. This study developed an integrated COC (ICOC) index to evaluate the outcomes of COC for multi-chronic patients. The benefits of this approach are as follows. First, the ICOC index integrated UPC, COCI, and SECOC, thus overcoming the limitations of a single index for different types of medical care. Second, ICOC can replace the individual COC index in interpreting a single condition. Its better model-fitting characteristics also implies that the ICOC index can avoid the bias often encountered in a single index. Third, the ICOC index can be widely applied to the evaluation of different chronic conditions or different types of research. It offers a reliable assessment of different levels of medical care and scopes of chronic conditions; it can also serve as an indicator in multilevel research.

For the measurement of COC and different levels of integrated care for multi-chronic patients, this study presents a preliminary integration of COC measurements and the investigation is limited to the association between COC and care outcomes. In short, the ICOC calculation is a new measurement method, which needs to accumulate more empirical research to establish a standardized measuring method. In the future, it can be widely applied to the evaluation of different chronic conditions or different types of research to verify the care outcome of diseases. In addition, future studies can consider an in-depth analysis of COC in relation to different scopes of medical care, such as by using the dose–response relationship or cut point analysis to find areas of improvement for COC. The findings may be beneficial to decision-makers in enhancing medical-seeking behavior and hospital integration outcomes.

This study does, however, certain limitations. First, although the medical system in Taiwan provides a superior environment for the evaluation of integrated health care, information regarding the degree of actual implementation and integration at individual medical facilities is unavailable. Second, some demographic characteristics and needs, which were not included in the variables, may affect the estimation of the COC outcomes. Third, even though most hospitals in Taiwan have trained staff handling the classification and coding of diseases, mistakes may still remain in the disease codes for the claims data used in this study. We consider this issue a systematic bias. Finally, it should be noted that there may be a correlation problem for the four aspects of COC measurements when using the same index. This is particularly true in the measurement of physician and medical site levels for the same chronic condition scope, as there may be a collinearity problem in constructing a linear model. In other words, when measuring a single disease, the COC measurement at physician level may be the same as that at medical site level. The method proposed in this study is thus not suitable for measuring the same disease. However, this study considers situations where there is a clear division of profession among specialties, and focuses on multi-chronic patients, so the correlation issue is almost negligible (we observed a low correlation in the data). In addition, as people in Taiwan are free to choose their medical facility and physicians, the proposed method may not apply to countries in which a referral system is the foundation of the medical system.

## Conclusions

The ICOC assessment model, which measures different scopes of chronic conditions at both medical facility level and physician level, has a better model fitting while overcoming the limited applications of single indices. As ICOC is an integrated index, more empirical evidence is required to establish a standardized measuring method for future research. Studies have shown that patients with higher COC have better outcomes at both medical facility level and physician level when the COC indexes are used to measure a major/single chronic condition. But, according to this study, poor care outcome is found when the treatment of all the diseases is concentrated at physician level. In other words, a patient with multi-chronic conditions receiving care services from the same physician may lead to poor care outcomes. This indicates that a patient-centered, multi-specialty-oriented health care structure for multi-chronic patients is important. Medical facilities should switch from a single-specialty service to multi-specialty integration or regional integration for multi-chronic patients to avoid medical errors and unnecessary medical costs. The findings of this study can serve as a reference for policy-makers and hospital administrators in terms of hospital management.

## Competing interests

The authors declare that they have no competing interests.

## Authors' contributions

CLC conceptualized the study, participated in the analysis of the data, drafted the manuscript and revised the manuscript. HTH conceptualized the study and participated in the study design, statistical analysis, and interpretation of data. HJY conceptualized the project, participated in the study design, interpretation of data, writing of the manuscript, and revised the manuscript. HWT integrated the results of the research into clinical problems and revised the manuscript. All authors have read and approved the final version of the manuscript.

## Pre-publication history

The pre-publication history for this paper can be accessed here:

http://www.biomedcentral.com/1472-6963/12/405/prepub

## Supplementary Material

Additional file 1 ICD-9-CM codes of chronic disease. Click here for file

Additional file 2 Range of disabilities. Click here for file
