# Supplementary material for: Using an integrated COC index and multilevel measurements to verify the care outcome of patients with multiple chronic conditions
Source: BMC Health Serv Res. 2012 Nov 19;12:405. doi: 10.1186/1472-6963-12-405 (PMC3529188; doi:10.1186/1472-6963-12-405)
Supplement: Additional file 1 — ICD-9-CM codes of chronic disease. [file 1472-6963-12-405-S1.pdf]

Additional files

Additional file 1 – ICD-9-CM codes of chronic disease

Chronic diseases	NHI chronic diseases classification codes	ICD-9-CM codes
• Diabetes	01	250.00-250.91
• Hypertension	02	402.00-402.91, 405.01-405.99
• Chronic hepatitis	03	571.0-571.9
• Liver cirrhosis	25	
• Chronic renal infection	04	580.0-589.9
• Thyroid dysfunction	05	240.0-246.9
• Asthma	06	490-493.91
• Chronic bronchitis	10	
• Emphysema	20	
• Peptic ulcer	08	531.00-533.91
• Arthritis	09	711.39-720.0, 725-729.9
• Heart disease	11	393-398.99, 410.00-410.92, 410.00-410.92, 427.0-427.9, 428.0-429.9
• Cancer	12	150.0-162.9, 163.0-176.9, 180.0-188.9, 189.0-189.9, 191.0-191.9, 192.0-201.07, 201.00-201.98, 204.00-208.91, 200.00-200.88, 202.00-203.81, 140.0-149.9, 230.0-234.9
• Drug treatment following organ transplantation	13	V63.0-V68.9
• Cerebrovascular disease	14	430-434.9, 436, 437.0
• Epilepsy	15	345.00-345.91
• Parkinson's disease	16	332.0-332.1
• Tuberculosis	17	010.00-016.96, 017.00-018.96
• Chronic cholangitis	18	534.00-537.9, 555.0-558.9, 565.0-570, 572.0-573.9, 575.9-579.9
• Gastrointestinal functional disorders	23	
• Hyperlipidemia	19	272.0-272.1
• Chronic obstructive pulmonary disease	21	495.0-496
• Lupus erythematosus	24	731.0-733.99, 737.0-739.9
• Osteoporosis	27	
• Raynaud's disease	26	441.0-443.9, 446.0-448.9
• Kawasaki disease complicated by cardiovascular abnormalitie	78	
• Psoriasis	28	690-709.9
• Systemic eczema	32	
• Blackfoot disease	79	

• Leukoplakia	83	
• Seborrheic dermatitis	84	
• Amyloidosis	85	
• Herpes dermatitis	86	
• Pemphigoid	87	
• Epidermal decomposition bullosa	88	
• Severity of ichthyosis	89	
• Keratosis follicularis	90	
• Progressive systemic scleroderma	91	
• Familial benign chronic pemphigus	92	
• Onychomycosis	29	110.0-118
• Gout	7	270.0-279.9, 251.0-259.9
• Pemphigus	30	
• Dermatomyositis	31	
• Hyperprolactinemia syndrome	43	
• Vice thyroid function hypothyroidism	52	
• Wilson's disease	53	
• Congenital metabolic disorders	48	
• Congenital or acquired immunodeficiency syndrome	70	
• Adrenal lesions caused by endocrine disorders	71	
• Pituitary lesions caused by endocrine disorders	72	
• Precocious puberty	80	
• Gonadal hypofunction	93	
• Glaucoma	33	365.00-365.9
• Xerophthalmia	34	375.00-375.9
• Retinal degeneration	35	360.00-364.9, 367.0-368.9,370.00-371.9, 372.4-374.9, 376.00-377.9, 379.00-379.99
• Macular degeneration	36	
• Uveitis	37	
• Vitreous hemorrhage	38	
• Corneal degeneration	39	
• Chronic anemia	40	280.0-285.9
• Purpura	41	286.0-289.9
• Persistent blood coagulation disorders (hemophilia)	63	
• Endometriosis	42	619.0-629.9
• Menopausal symptoms	67	
• Inner ear vestibular lesion	44	384.00-388.9,380.00-380.9
• Chronic sinusitis	45	472.0-473.9
• Chronic media otitis	46	381.00-383.9
• Psychosis	47	290.0-301.9
• Muscle rigidity dystrophy	49	330.0-336.9

• Other central nervous system deterioration and genetic diseases	54	
• Polymyositis	50	710.0-713.8,715.00-716.99
• Myasthenia gravis	51	323.0-326, 337.0-342.9, 346.00-359.9
• Multiple sclerosis	55	340
• Infant with cerebral palsy and other paralytic syndrome	56	343.0-344.9
• Atherosclerosis<br>• Arterial embolism and thrombosis	57<br>58	440.0-440.9
• Bronchiectasis<br>• Pneumoconiosis<br>• Lung disease due to external agents	22<br>59<br>60	500-508.9
• Kidney infection	61	590.0-590.9
• Congenital deformities	62	740.0-742.9, 745.0-751.9, 754.30-756.9, 743.00-744.9, 752.0-753.9, 757.0-759.9
• Leprosy	64	030.0-030.9
• Hemorrhoids	65	455.0-455.9
• Prostatic hypertrophy	66	600
• Urinary incontinence	68	780.0-787.9, 788.1, 788.3-796.9, 798.1-799.0, 799.2-799.9
• Oil syndrome of PCBs poisoning	69	905.0-909.9
• Brain tumors concurrent nerve dysfunction	73	225.0-225.9
• Multiple peripheral nerve dysfunction<br>• Plexopathy<br>• Trigeminal neuropathy<br>• Spinal injuries<br>• Migraine	74,<br>75<br>76<br>77<br>81	323.0-326, 337.0-337.9, 341.0-342.9, 346.00-359.9
• Allergic rhinitis	82	475-478.9
• Chronic prostatitis (confirmed by proactive prostatic fluid secretion taken from prostate massage)	94	601.0-602.9, 604.0-604.99, 607.0-608.9
• Chronic osteomyelitis	95	730.00-730.99
• Myelodysplastic syndrome	96	284.9,285.0,205.2,250.8,206.1,238.7
• Idiopathic thrombocytopenic hyperplasia	97	238.7
• Chronic urticaria	98	708
