# Supplementary material for: Using an integrated COC index and multilevel measurements to verify the care outcome of patients with multiple chronic conditions
Source: BMC Health Serv Res. 2012 Nov 19;12:405. doi: 10.1186/1472-6963-12-405 (PMC3529188; doi:10.1186/1472-6963-12-405)
Supplement: Additional file 2 — Range of disabilities. [file 1472-6963-12-405-S2.pdf]

Additional file 2 – Range of disabilities

1. Visually impaired, hearing impaired, balance dysfunction, sound dysfunction or language dysfunction
2. Physical disabilities
3. Mentally retarded
4. Vital organs lose their function: liver, lung, kidney, stomach, intestinal tract, bladder and hematopoietic function disorders, swallowing dysfunction
5. Facial injuries
6. Vegetative state
7. Dementia
8. Autism
9. Multiple disabilities
10. Patients with chronic mental illness, stubborn (refractory) epilepsy, or rare diseases identified by the central health authorities to cause impairments in physical and mental functions
11. Others: chromosomal abnormalities, congenital metabolic diseases, other birth defects
